# Transcriptomes of microglia in experimental cerebral malaria in mice in the presence and absence of Type I Interferon signaling

**DOI:** 10.1186/s13104-018-4020-3

**Published:** 2018-12-20

**Authors:** Carlos Talavera-López, Barbara Capuccini, Richard Mitter, Jing-wen Lin, Jean Langhorne

**Affiliations:** 1 Malaria Immunology Laboratory, The Francis Crick Institute, 1 Midland Road, London, NW1 1AT UK; 2 Present Address: Polo di Genomica Genetica e Biologia, Via Fiorentina 1, 53100 Siena, Italy; 3 Present Address: Division of Pediatric Infectious Diseases, State Key Laboratory of Biotherapy, West China Second Hospital, Sichuan University and Collaboration Innovation Centre, Chengdu, China; 4 Bioinformatics and Biostatistics team, The Francis Crick Institute, 1 Midland Road, London, NW1 1AT UK

**Keywords:** Malaria, Microglia, *Plasmodium berghei*, Experimental cerebral malaria, Type I Interferons, Microarray

## Abstract

**Objectives:**

*Plasmodium berghei* ANKA infection in mice is a model for human cerebral malaria, the most severe complication of *Plasmodium falciparum* infection. Responses of brain microglia have been little investigated, and may contribute to the pathogenesis of cerebral malaria. We showed previously that microglia are activated in *P. berghei* infections, and that Type 1-Interferon signaling is important for activation. This dataset compares transcriptomic profiles of brain microglia of infected mice in the presence and absence of Type 1 interferon signaling, with the aim of identifying genes in microglia in this pathway during experimental cerebral malaria.

**Data description:**

We documented global gene expression from microglial RNA from uninfected and *P berghei*-infected wild-type C57BL/6 and IFNA Receptor Knock-out mice using Illumina Beadarrays. Principal component analysis showed 4 groups of samples corresponding to naïve wild-type, naïve IFNA Receptor knock-out, infected wild-type, and IFNA Receptor knock-out mice. Differentially-expressed genes of microglia from the two groups of infected mice are documented. Gene set enrichment analysis showing the top 500 genes assigned to Reactome pathways from infected IFNA Receptor knock-out versus naïve, and infected WT versus naïve has been generated. These data will be useful for those interested in microglia cells, and in experimental cerebral malaria.

## Objective

Cerebral Malaria is one of the most severe complications of infection with the malaria parasite *Plasmodium falciparum*. A widely used animal model to investigate pathogenic processes in cerebral malaria, and the contribution of the host response, is the C57BL/6 mouse infected with the ANKA strain of *Plasmodium berghei* (experimental cerebral malaria, or ECM, [1]). We showed previously that microglia are activated in the brains of mice infected with *P. berghei* ANKA undergoing ECM. At the time of ECM symptoms, immune response and chemokine genes were significantly upregulated. Gene Ontology analysis and functional gene enrichment suggested that these responses were driven by Type I interferons [2]. In support of this, we showed that IFNβ activated microglia in vitro to produce those chemokines, whose gene expression was upregulated in the microarray analysis [2]. As Type 1 IFN signaling can have different roles in malaria infections it would be important to determine the contributions of signaling through the Type I IFN receptor on microglia in ECM, and thus whether microglia play any part on the pathogenesis, or control of pathology, in ECM, which might have implications for human disease. We wanted to investigate the possible effects on microglia and ECM of abrogating signaling through the IFN-1 receptor. In the analysis shown in this Data Note, we compared the transcriptome of purified microglia from *P. berghei*-infected IFNA Receptor knock-out mice with that of infected wild-type C57BL/6 mice using Illumina Beadarrays.

An analysis of gene expression microglia in wild-type C57Bl/6 mice has been published [2], but a comparative study with microglia from IFNA Receptor knock-out mice has not been documented previously. These microarray results have not been incorporated into a research publication as we could not continue this study in this form as the Illumina Beadarrays have been discontinued. Nevertheless, we believe these data may be a useful for those research groups interested in activation of microglia in various settings and for malaria immunologists interested in the mechanisms of ECM in this rodent model.

## Data description

We collected gene expression data from microglia isolated from wild-type C57Bl/6 mice and IFNA Receptor knock-out mice infected with *Plasmodium berghei* ANKA using Illumina Beadarrays (Table 1, data file 1).

**Table 1:** Overview of data files/data sets

Label	Name of data file/data set	File types (file extension)	Data repository and identifier (DOI or accession number)
*Data file 1*	Microarray data C57Bl/6 mice infected *with P. berghei*	*Illumina idat*	https://www.ncbi.nlm.nih.gov/geo/query/acc.cgi?acc=GSE119650
*Data file 2*	PCA.pdf	*pdf*	https://figshare.com/s/37fc3835ea53be4bf94f
*Data file 3*	wt.inf_vs_wt.naive-p01.fc2.results.txt	*txt*	https://figshare.com/s/37fc3835ea53be4bf94f
*Data file 4*	ko.inf_vs_ko.naive-p01.fc2.results.txt	*txt*	https://figshare.com/s/37fc3835ea53be4bf94f
*Data file 5*	ToppGene.enrichment.barplot.pdf	*pdf*	https://figshare.com/s/37fc3835ea53be4bf94f
*Data file 6*	ko_specific_treatment_effect.heatmap.pdf	*pdf*	https://figshare.com/s/37fc3835ea53be4bf94f

Female C57BL/6 mice and mice lacking the IFNA Receptor (IFNARKO) (aged 6–9 weeks of age) were obtained from the SPF breeding unit of the Mill Hill Laboratory, Francis Crick Institute, and for experimental work were conventionally housed with sterile bedding, food and irradiated water. Room temperature was 22 °C with a 12 h light/dark cycle; food and water were provided ad libitum. Mice were injected with 10^5^ *P. berghei* ANKA infected erythrocytes intraperitoneally. Mortality, parasitemia and clinical scores indicative of ECM were monitored daily. Naïve and day 7-infected (d7) infected WT and IFNARKO mice were euthanised using pentobarbital, injected intraperitoneally (600 mg/kg body weight). Isolation of microglia is described in detail in [2]. Briefly, microglia were isolated from the brains of uninfected C57Bl/6 and IFNARKO mice and from both groups of infected mice. Microglia (CD45l^ow^ and CD11b^+^) were purified from other brain cells by flow cytometry (MoFlo XPD, Beckman Coulter) using a combination of fluorophore conjugated antibodies: APC-anti-CD11b, PE-CD45, APCCy7-Ly6C, pacific blue- -H-2 Kb (Biolegend). Cells were washed and resuspended in PBS containing 2% FCS. Analysis was carried out using FlowJo-X software (Treestar). The sorted cells were confirmed as microglia based on the lack of cell surface marker Ly6C.

Total RNA was extracted immediately after sorting from approximately 10^5^ microglial cells using Ribopure kit (Ambion), and concentrations determined by Qubit quantitation using the HS assay kit (ThermoFisher Scientific). Quality was assessed by the Agilent 2100 Bioanalyzer; samples with a RIN score above 8.50 were used. Total RNA (300 ng) of each sample was amplified using the Total prep RNA amplification kit (Illumina) and Amplified cDNA (1500 ng) were then hybridized to Illumina MOUSE WG-6 V2.0 Beadarrays at 58 °C for 14–20 h at the High Throughput Screening facility of the Francis Crick institute RNA and cDNA were quantified by Qubit fluorometric quantitation and the quality were analysed using Agilent 2100 Bioanalyzer at each step ([2] and Table 1, Data file 1)

Data analysis was conducted using the *limma* package [3] within R v3.5.1 running Bioconductor v3.7. Illumina idat files were read using “*read.idat*” function together with manifest file MouseWG-6_V2_0_R3_11278593_A.bgx downloaded from the Illumina website. Detection *p*-values were calculated using the “*detectionPValues*” function with default settings. Background correction was performed using negative control probes followed by quantile normalization using negative and positive control probes via the “*neqc*” function. Normalised expression values are reported in a log_2_ scale.

Principal Component Analysis was performed on the 500 genes showing greatest variance across samples (Table 1, Data file 2).

Differential gene expression was assessed between infected and naïve cell states within KO and WT cells separately using a linear model (Table 1, Data files 3 and 4). Significance was determined using a threshold based on a FDR ≤ 0.01 together with an absolute fold change ≥ 2.

The two resulting lists were ordered by absolute fold change and the top 500 unique Entrez gene identifiers from each were put forward for gene list enrichment analysis using the ToppGene Suite [4]. Hits to the Reactome [5] pathway (FDR ≤ 0.01) are presented in the barplot (Table 1, Data file 5).

A nested interaction formula was used to select genes responding differently to infection between KO and WT cells. Genes showing a KO specific response but remain unchanged in WT cells were selected for visualisation in a heatmap (Data file 6). Each gene’s expression across samples was converted to a z-score to aid visualisation. Clustering of row and columns was conducted using complete linkage on a Euclidean distance matrix.

## Limitations

- The sample size for each group is relatively small, 5 mice per group.
- There is only one time-point in the infection.

It was intended to continue this study; however, Illumina ceased production of these arrays during the study, and therefore the experiment could not be extended using this microarray format.

## Abbreviations

ECM: experimental cerebral malaria
IFNARKO: Interferon Alfa Receptor 1 knock-out
iRBC: infected red blood cells
KO: knock out
*P. berghei*: *Plasmodium berghei*
WT: wild-type
