# Correction to: Transcriptomes of microglia in experimental cerebral malaria in mice in the presence and absence of Type I Interferon signaling

**DOI:** 10.1186/s13104-019-4069-7

**Published:** 2019-01-22

**Authors:** Carlos Talavera-López, Barbara Capuccini, Richard Mitter, Jing-wen Lin, Jean Langhorne

**Affiliations:** 1 Malaria Immunology Laboratory, The Francis Crick Institute, 1 Midland Road, London, NW1 1AT UK; 2 Present Address: Polo di Genomica Genetica e Biologia, Via Fiorentina 1, 53100 Siena, Italy; 3 Present Address: Division of Pediatric Infectious Diseases, State Key Laboratory of Biotherapy, West China Second Hospital, Sichuan University and Collaboration Innovation Centre, Chengdu, China; 4 Bioinformatics and Biostatistics Team, The Francis Crick Institute, 1 Midland Road, London, NW1 1AT UK

## Correction to: BMC Res Notes (2018) 11:913 10.1186/s13104-018-4020-3

Following publication of the original article [1], an error was reported in Table 1. The data repository links in the 4th column were incorrect. In this Correction, the corrected version of Table 1 is shown. The original publication of this article has been corrected.

**Table 1:** Overview of data files/data sets

Label	Name of data file/data set	File types (file extension)	Data repository and identifier (DOI or accession number)
*Data file 1*	Microarray data C57Bl/6 mice infected *with P. berghei*	*Illumina idat*	https://www.ncbi.nlm.nih.gov/geo/query/acc.cgi?acc=GSE119650
*Data file 2*	PCA.pdf	*pdf*	10.6084/m9.figshare.7171952
*Data file 3*	wt.inf_vs_wt.naive-p01.fc2.results.txt	*txt*	10.6084/m9.figshare.7171952
*Data file 4*	ko.inf_vs_ko.naive-p01.fc2.results.txt	*txt*	10.6084/m9.figshare.7171952
*Data file 5*	ToppGene.enrichment.barplot.pdf	*pdf*	10.6084/m9.figshare.7171952
*Data file 6*	ko_specific_treatment_effect.heatmap.pd	*pdf*	10.6084/m9.figshare.7171952

The publisher apologises to the authors and readers for the inconvenience.
